# Understanding Electrical Failure of Polyimide-Based Flexible Neural Implants: The Role of Thin Film Adhesion

**DOI:** 10.3390/polym14183702

**Published:** 2022-09-06

**Authors:** Marcel Tintelott, Andreas Schander, Walter Lang

**Affiliations:** 1 Institute for Microsensors, Actuators, Systems (IMSAS), University of Bremen, Otto-Hahn-Allee 1, 28359 Bremen, Germany; 2 Institute of Materials in Electrical Engineering 1 (IWE 1), RWTH Aachen University, Sommerfeldstraße 24, 52074 Aachen, Germany

**Keywords:** chronic stability, bioelectronics, thin film passivation, neural probe

## Abstract

The lack of long-term stability of polymeric neural interfaces remains one of the most important and less tackled issues in this research field. To address this issue, we fabricated two test structures based on interdigitated electrodes (IDEs) encapsulated with polyimide (PI). One of the test samples was pretreated with barrel oxygen plasma prior to spin coating of the second PI layer. The second test structure was pretreated using a reactive ion etching (RIE) process. The test samples were immersed in an electrolyte solution at elevated temperatures to mimic the conditions inside the human brain. The samples were then electrically and mechanically stressed to accelerate their degradation. Real-time monitoring of the electrical insulation stability was used to compare the impact of the pretreatment on the long-term stability. Barrel-plasma-activated test samples showed a mean lifetime of 1.5 days, whereas RIE pretreatment increased the mean lifetime to 24 days. Therefore, RIE-pretreated test samples exhibited 16 times longer mean stability compared to purely chemically activated test samples. Furthermore, the electrical measurements were correlated with mechanical adhesion tests. Chemically activated test samples showed significant delamination, whereas RIE pretreatment enhanced the adhesion, and no delamination could be observed. The correlation of these investigations suggests that the adhesion between different layers is higher following RIE pretreatment compared to pretreatment with chemical barrel plasma. In conclusion, the adhesion between the two PI foils seems to play a key role in the long-term stability of such devices.

## 1. Introduction

Since the Italian scientist Luigi Galvani published the first work on the electrical nature of the nervous system, many scientists have attempted to understand the mechanism of communication between individual neurons [1,2,3]. With the advent of micro- and nanofabrication, neuroscientists and engineers attempted to develop neural interfaces to record the neural activity of single neurons over long periods of time. Long-term measurements of the neural activity of single neurons could provide useful data to understand aging and learning mechanisms [4,5,6]. Since Wise et al. revealed the first recording of neural signals with silicon-based Michigan probes, many studies have been published showing improved recording quality (i.e., a higher signal-to-noise ratio (SNR)) of an increasing quantity of electrodes within a single device, allowing for high-resolution mapping of neural activity [7,8,9,10]. Since then, neural probes have evolved from silicon-based rigid neural probes to polymer-based flexible neural probes with various device architectures [11,12,13,14,15,16]. The advantage of polymer-based neural interfaces is the possibility of reducing damage to the tissue and protecting interior signal tracks against ionic body fluids. The use of various polymeric materials (e.g., polyimide (PI) or parylene C) is often reported in the literature for the fabrication of flexible neural probes [17,18,19,20]. Parylene C is a favorable material due to its biocompatibility, pinhole-free nature, and its easy processing via room-temperature chemical vapor deposition (CVD) [21]. PI is also a promising material for the fabrication of chronical flexible neural implants. It can be easily coated via spin-coating technique and is, therefore, fully cleanroom-compatible. In comparison to CVD deposition of parylene C, a PI layer can be obtained within a reduced amount of time. Furthermore, considerable achievements have been reported with respect to PI implants. Waschkowski et al. reported the fabrication and characterization of a large electrode array for epiretinal stimulation [22]. In a follow-up, good biocompatibility of PI-based epiretinal implants with integrated silicon sensor chips was demonstrated [23]. In addition, wireless PI-based epiretinal implants were implanted in the human eye during a clinical trial [24]. Besides the electrical performance of PI-based implants, their long-term stability is a critical factor. The goal of chronically long-term stability is an important consideration with respect to improving the performance and lifetime of neural implants. However, the cause of the failure of such devices is often neglected in the scientific community [25]. Failures of such devices are often reported as “high-impedance” electrodes or “electrode breakage” [25]. Understanding how and why neural probes fail is of considerable importance for the fabrication and development of more stable devices and achieving the goal of clinical viability (continuous functionality for ten years or more in vivo).

In previous work, we demonstrated a novel in vitro method to investigate the long-term stability of neural probes [26]. Test samples were stored in saline solution at elevated temperature in combination with high-voltage stress. In addition, periodic mechanical stressing was used to accelerate the degradation process. In this work, we fabricated two test samples with different pretreatments prior to the deposition of the top encapsulation layer and investigated the cause of early-stage failure. Therefore, we utilized our previously developed method to induce electrical failure of the test samples. Optical microscopy was used to investigate the origin of the electrical failure. In addition to the electrical investigation, cross-cut tests were performed to investigate the adhesion between the individual layers. The cross-cut tests were evaluated using optical microscopy.

## 2. Materials and Methods

### 2.1. Sample Fabrication

Test samples were designed and fabricated using standard cleanroom processes, which are used for the fabrication of state-of-the-art flexible neural probes. The test samples were designed as interdigital structures embedded between two 5 µm thick PI layers on a 100 mm silicon wafer. Furthermore, test samples based on parallel metal traces were designed for the investigation of mechanical stability.

**Figure 1:**
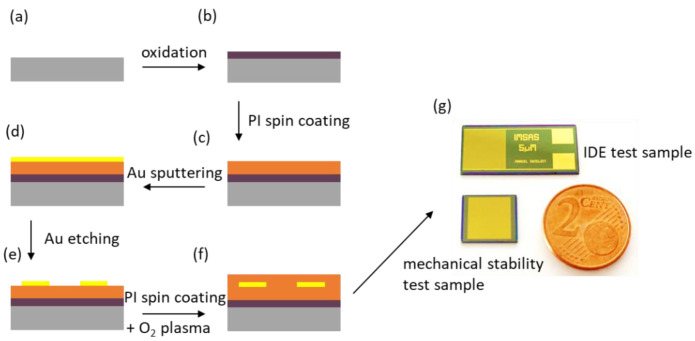
Schematic representation of the fabrication flow of the test samples: A silicon wafer served as a starting material (**a**). To ensure proper electrical insulation of the substrate, the silicon wafer was oxidized (**b**). After applying the adhesion promoter, a 5 µm thick PI layer was spin-coated (**c**). A 300 nm thick Au layer was deposited using sputter deposition (**d**). Subsequently, a photolithography process was carried out, and the Au layer was structured via wet chemical etching (**e**). Different pretreatments were used immediately before the spin coating of a second PI layer (**f**). Photograph of the two test structures (**g**).

A schematic representation of the fabrication flow used to fabricate the test structures is shown in Figure 1. First, thermal oxidation was used to grow a 500 nm thick silicon oxide structure as an insulation layer between the silicon substrate and the test sample (Figure 1a,b). To ensure strong adhesion between the first PI layer and the silicon oxide, adhesion promoter 3-aminopropyltriethoxysilane (0.1 vol. %, Sigma-Aldrich Chemie GmbH, Taufkirchen, Germany) was used. As shown in Figure 1c PI U-Varnish-S (UBE Europe GmbH, Germany) was spin-coated at 3000 rpm directly after treatment with the adhesion promoter and cured as described in previous works using a vacuum hotplate (UniTemp GmbH, Pfaffenhofen/Ilm, Germany) [11,26,27]. A gold (Au) layer with a thickness of 300 nm was sputtered (100 W, DC magnetron) directly on the PI layer (see Figure 1d). A 1.8 µm thick AZ1518 positive-tone photoresistor (MicroChemicals GmbH, Ulm, Germany) was processed using standard photolithography processes. Afterward, the Au layer was structured using Au Etch 200 solution (NB Technologies GmbH, Bremen, Germany), and the photoresistor was removed using AZ 100 remover (MicroChemicals GmbH, Ulm, Germany) (see Figure 1e). Immediately prior to spin-coating of the second PI layer, two different oxygen plasma pretreatments were applied. On the one hand, a chemical oxygen plasma treatment (barrel plasma, 30 s, 500 W, 500 sccm O_2_) was carried out immediately prior to spin coating and curing the second PI layer. On the other hand, a reactive ion etching (RIE) process using an inductively coupled plasma tool (STS Multiplex ICP, Surface Technology Systems GmbH, Ulm, Germany, 30 s, 800 W coil, 25 W bias, 40 sccm O_2_, 5 mTorr) was used as a second pretreatment method. Immediately after these pretreatment steps, the second PI layer was spin-coated and cured as before, yielding a second 5 µm thick PI layer (Figure 1f). Finally, the PI was removed in the contact-pad areas and dicing regions using an O_2_/CF4 reactive ion etching process. Therefore, a 20 µm thick AZ 9260 photoresistor (MicroChemicals GmbH, Ulm, Germany) was processed using standard photolithography processes. Figure 1g shows a photograph of the final fabricated IDE test sample and the mechanical stability test sample.

### 2.2. Long-Term Stability Testing

To identify the reasons for electronic failure, we used our previously published method to degrade the fabricated test samples [26]. The soak test is a typical experimental setup used to investigate the response of neural interfaces exposed to liquids/electrolytes [27,28,29,30]. The electrical test samples were stored in Ringer’s solution (B. Braun Melsungen AG, Melsungen, Germany) at an elevated constant temperature of 70 °C to enhance the diffusion of the electrolyte into the polymeric encapsulation. A hotplate was used to heat up the electrolyte. The faster diffusion rate of saline combined with a higher temperature will result in faster degradation of the polymeric test sample. To electrically stress the test sample, a 10 V DC voltage was applied between the two electrodes of the test sample with a 1 kΩ resistor in series. The voltage drop across the serial resistor was measured in real-time with a sampling rate of 1 Hz. A voltage threshold (in this study, 1 V) can be set to classify the test sample as failed, as demonstrated in our previous work. Here, we utilized the real-time signal to extract knowledge about the defect trend of PI-based insulation layers. Furthermore, mechanical stress was applied to the test samples using ultrasonic treatment (35 kHz, 300 W power). The ultrasonic treatment was performed every two weeks for 30 min at room temperature.

### 2.3. Cross-Cut Test

Cross-cut tests were performed using a commercially available cross-cut tool (Gitterschnittgerät CC1000 6 × 1 mm, mtv messtechnik oHG) with a cutting distance of 1 mm. This test was manually performed with minimal force. The cutting was aligned with the metal structures on the cross-cut test samples. The first cut was performed at a 90° angle to the metal structures, whereas the second cut was performed parallel to the metal structures. Optical microscopy was used to evaluate the cross-cut test. Here, the test criterion was the delamination of the top PI layer.

### 2.4. Impedance Spectroscopy

Impedance spectroscopy was used to determine the water uptake of PI to identify the impact of water absorption on long-term stability. Therefore, the fabricated test samples were exposed to Ringer’s solution at an elevated temperature of 70 °C. The impedance of the interdigital electrode (IDE) structure was monitored for 100 h. The spectra were recorded from 1 to 10 kHz. The impedance at a frequency of 10 Hz was used to evaluate the impedance changes of the test samples.

### 2.5. Finite-Element-Method Simulation

**Figure 2:**
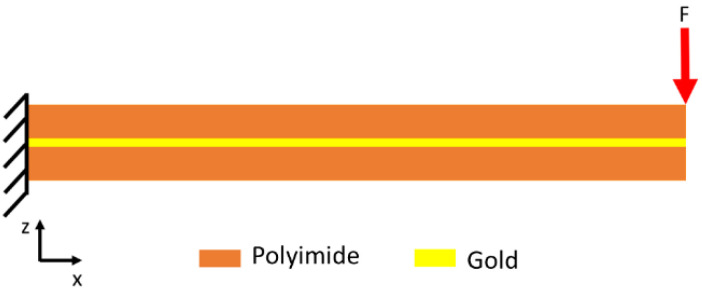
Schematic illustration of the mechanical model used to investigate the mechanical stress occurring as a result of bending.

In a flexible neural implant, tension and bending stress can occur due to the difference in the elastic moduli of the used materials. If the flexible leads of an implanted electrical interface are imagined as a clamped beam, tensile and bending loads can act on the implant. These loads cause mechanical stresses in the metallization and encapsulation. Because the metallization is much thinner than the encapsulation, the metallization has to travel the same distance as the encapsulation. The deformation behavior of linear elastic materials is described by Hook’s law. To gain insights into the mechanical stress occurring between the PI encapsulation and the metal traces, a 2D finite-element-method (FEM) simulation was carried out. In this simulation, a PI/Au/PI stack was used as a cantilever to investigate the stress inside the PI-encapsulated Au traces due to bending. The model was implemented as shown in Figure 2. Here, one side of the structure was fixed, and the force was applied to the moveable side of the material system. The simulation was carried out using Comsol Multiphysics.

## 3. Results and Discussion

### 3.1. What Can Affect Long-Term Stability?

**Figure 3:**
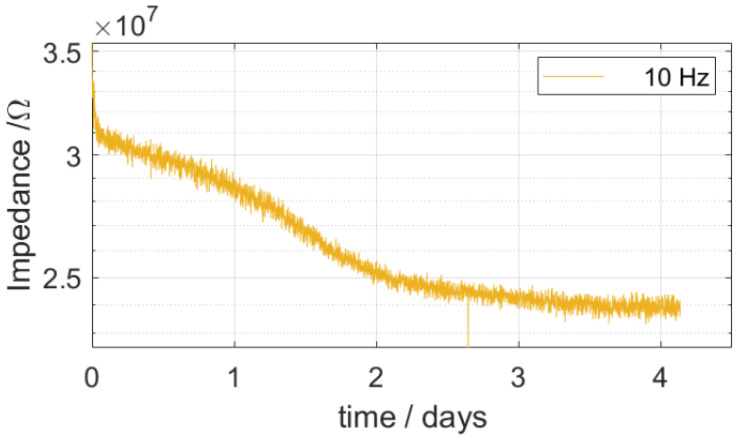
Real-time monitored impedance (at 10 Hz) during a soak test at an elevated temperature of 70 °C.

To understand the early-stage failure of polymer-based neural probes, several investigations were performed to identify the origin of electrical failure. First, transient impedance spectroscopy measurements were performed to monitor the diffusion of water into the PI encapsulation layer. A soak test was performed at an elevated temperature of 70 °C to enhance the diffusion of the electrolyte solution into the PI encapsulation. Figure 3 shows the impedance (at 10 Hz) over time. The impedance decreased the most within the first two days and continued to decrease linearly over the course of the third day before stabilizing. Therefore, it can be concluded that the water uptake of the PI reaches its maximum after around 3 days.

In a previous study, we showed that samples without any surface modification exhibited a lifetime of 4 ± 3.8 days. In contrast to these results, a PI layer covering IDEs on a solid glass substrate resulted in electrical insulation stability for more than 170 days [31]. In the latter study, a soak test was performed at elevated temperatures (60 °C), and the insulation stability was investigated using impedance spectroscopy. Comparing these two results reveals that active electrical and mechanical stress results in much faster degradation of insulation stability. Therefore, interface adhesion seems to play a key role in the long-term stability of polymeric encapsulations. The diffusion of water into the polymeric encapsulation may play a crucial role in the lifetime of the electrical encapsulation of neural probes. The diffusion of water molecules can lead to delamination of the layers of a flexible neural probe due to the weakening of polar attractions between non-covalently bonded layers or mechanical interlocking [25]. However, delamination can result in the formation of unwanted electrically conductive paths or cracking of the metal layers, resulting in the failure of such devices.

**Figure 4:**
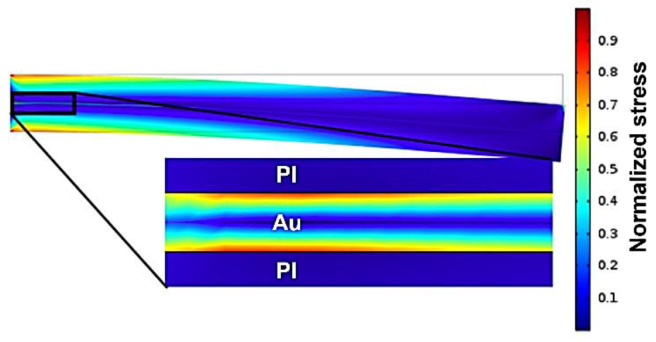
FEM simulation of normalized mechanical stress occurring in a PI/Au/PI material system.

Delamination does not only result from the diffusion of water into the polymeric encapsulation but can also originate from mechanical stress. Implanted probes are exposed to the mechanical micromotion of the brain [32]. Although metal traces are placed on the neutral axis, bending of the flexible neural implant creates stress at the interface of the metal traces and the polymeric encapsulation layer. Our group fabricated several generations of free-floating neural probes [11,12]. Besides recording the neural activity of rats, various methods were used to evaluate the mechanical stability of the fabricated neural probes. One simple method to evaluate mechanical stability is based on simple manual bending tests using a tweezer. This simple bending test showed clear differences between barrel-plasma- and RIE-pretreated samples. No delamination could was observed in RIE-pretreated samples, whereas 9 out of 10 barrel-pretreated samples exhibited notable delamination [33]. Therefore, a clear improvement in the mechanical stability was observed after RIE pretreatment. A simple cantilever-based FEM simulation was carried out to mimic the manual bending process. The goal of this simulation was to obtain a qualitative understanding of the resulting stress at the metal/polymer interface near the fixed point. Because it is difficult to determine the actual applied force during a manual bending procedure, the resulting stress within the material system was normalized for analysis. Figure 4 shows the normalized results of an FEM simulation for the stress analysis of polymeric encapsulated metal traces. Here, the bending of such material systems can result in high stress at the metal/polymer interface due to differences in elastic modulus. The simulation showed that the bending of flexible neural probes created high stress at the metal/polymer interface and thus, can result in delamination of the polymeric encapsulation layer [33]. The mechanical stress can be reduced by decreasing the thickness of the metal traces, resulting in increased electrode impedance.

### 3.2. Electrical Stability

The test samples were stressed until failure using our previously reported method [18]. To accelerate the degradation of the test samples, they were stored at an elevated temperature (70 ° C) and mechanically stressed in an ultrasonic bath for 30 min every 14 days and immediately prior to electrical failure monitoring. The test samples were connected to a serial resistor, and a voltage of 10 V was applied to the circuit. The leakage current was obtained by measuring the voltage drop across a serial resistor. For a proper encapsulated test sample, a voltage drop of 0 V is expected due to the high resistance (ideally infinite) of the capacitive test structure. The passivation capability of the polymeric encapsulation can be characterized by measuring the voltage drop across the serial resistance.

**Figure 5:**
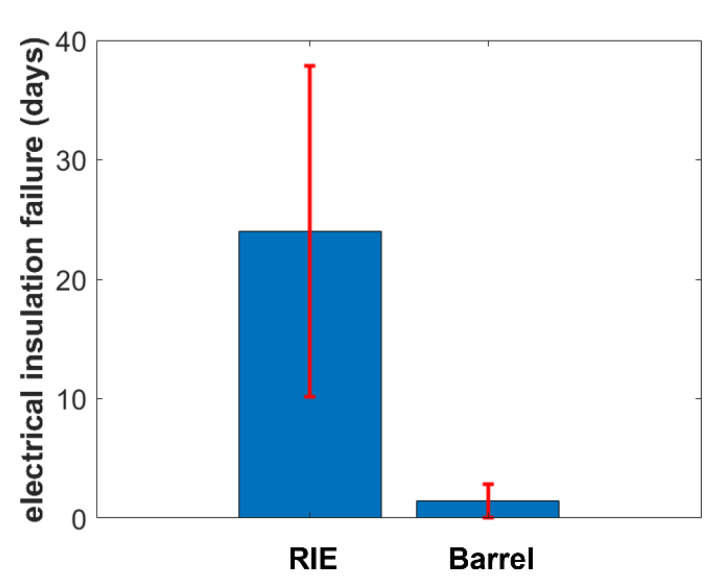
Comparison of the electrical insulation failure of RIE- and barrel-oxygen-plasma-pre-treated samples (*n* = 4).

Figure 5 shows the resulting mean early-stage insulation failure of the test samples pretreated with barrel oxygen plasma and RIE. The end of a lifetime was defined as the moment at which the voltage drop was equal to or higher than 1 V. Here, RIE-pretreated test samples exhibited longer mean stability compared to barrel-oxygen-plasma-pretreated samples. The pretreatment using a barrel oxygen plasma resulted in early-stage failure after around 1.5 days, whereas failure of RIE-pretreated test samples started after a mean of around 24 days. Therefore, RIE pretreatment results in 16 times longer mean stability of PI encapsulated signal traces than barrel pretreatment.

**Figure 6:**
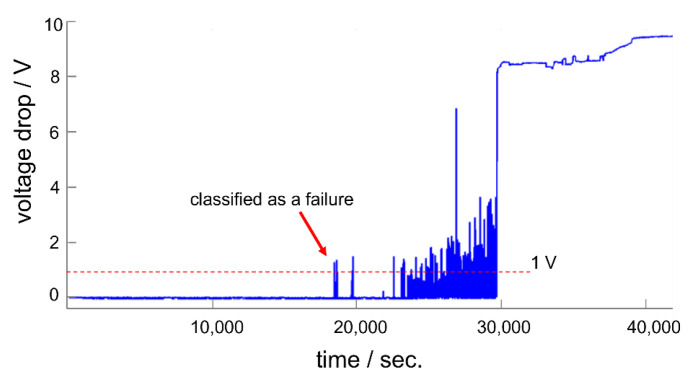
Real-time monitoring of the insulation stability of a barrel-plasma-pretreated test sample.

A relatively large standard deviation was observed for RIE-pretreated test samples. However, this standard deviation was not the result of an unreproducible stability testing scheme but was related to the test sample itself. A closer look at the real-time monitored data provides more insights. Figure 6 shows exemplary real-time monitoring data of the voltage drop across a test sample (a barrel-plasma-pretreated test sample). Single spikes in the real-time signal were observed around 5.5 h after the measurement started. As we defined failure of long-term stability as a voltage drop of 1 V, such spikes caused the failure of the encapsulated system. According to these spikes, the failure of PI-encapsulated signal traces is a dynamic process. At the beginning of this process, single spikes occurred, and with increasing time and voltage stress, the number of spikes increased until complete system failure.

The large standard deviation is partly based on these spikes. Single spikes can occur with a large time gap (up to days) before further spikes are visible. These spikes are the result of the formation of conductive paths, which are formed by an electrolyte bridge between the two electrodes. However, optical microscopy did not show any electrochemical reaction results at the electrodes. Therefore, we were not able to investigate the reason behind these spikes. Furthermore, the large standard deviation provides us with information about the homogeneity of the process itself. In the case of a perfect process, all test structures would fail around the same time with a minimum standard deviation. As shown in this work, the use of a plasma pretreatment seems to result in varied adhesion properties across the wafer. We also observed a large standard deviation in a previous study [26]. Therefore, the reason for the large standard deviation needs to be elaborated further to improve the encapsulation stability of polymeric encapsulated neural interfaces. Although the test samples exhibited a large standard deviation, we clearly demonstrated that RIE pretreatment enhances the long-term stability of PI-based neural interfaces.

### 3.3. Interface Adhesion

**Figure 7:**
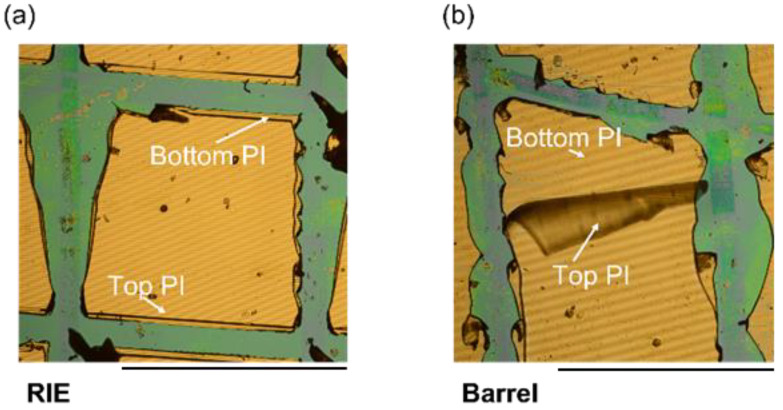
An exemplary result of the cross-cut test for an RIE-pretreated sample (**a**) without significant delamination and a barrel-pretreated sample (**b**), which exhibited significant delamination of the top PI encapsulation layer. Scale bars = 1 mm.

The failure of the electrical insulation of flexible neural probes is a serious limitation when it comes to real medical applications [25]. Therefore, we investigated the mechanical stability of the two test samples using cross-cut tests to obtain insights into the reasons behind electrical insulation failure. Here, a commercially available cross-cut tool was used. Cut lines were drawn at an angle of ~90°. As shown in Figure 7, the RIE sample exhibited no significant delamination due to mechanical stressing. The top PI layer remained on top of the metal/PI layer, whereas the barrel-pretreated sample showed complete delamination. The mechanical stability of the two samples matched the results of the electrical insulation stability of the test samples. Our group previously showed that the barrel-plasma-treated PI layer exhibited a peel strength of 29.3 mN/mm, whereas RIE pretreatment resulted in an adhesion strength higher than the mechanical resistance of the PI film [34]. Although barrel-plasma-treated PI films exhibited similar surface energy compared to RIE-pretreated PI films, a significant difference in adhesion strength was observed. These results could be the result of increased roughness of RIE pretreated PI films compared to barrel-plasma-pretreated films. Increased roughness increases the surface area and thus the physical attraction between two PI films and could explain the increased adhesion. The increase in surface roughness of PI layers after RIE pretreatment was previously demonstrated by other groups [35,36,37,38,39,40]. Here, atomic force microscopy (AFM) was used to determine the RMS surface roughness after RIE. A possible explanation for the increased surface roughness is the difference in the etching rate of crystalline and amorphous regions of the PI layer. Kim et al. showed that the RMS surface roughness could be increased from 7.7 nm to 180 nm after an RIE process [40]. Furthermore, it was shown that the chemical composition of PI changes as a result of oxygen plasma treatment. As demonstrated by multiple groups, an increased concentration of O-C and O=C bonds can be observed by X-ray photoelectron spectroscopy (XPS) after treating PI with oxygen plasma [35,36,37,38,39,40]. The increase in O-C and O=C bonds increases the O/C ratio of the PI surface. Furthermore, it is possible that the imide ring is opened during the RIE process, which could result in the formation of polyamic acid or PI precursor. As a result of the formation of radicals during the pretreatment process, functional groups such as -OH or -COOH could be formed. Such functional groups can additionally enhance chemical interactions with the second spin-coated PI precursor. Moreover an excessive etching time or excessive power can result in etching of the chemically active layer of the PI layer. Furthermore, we investigated the impact of an autoclave sterilization process on the mechanical stability of the two test samples. Here, no significant differences were observed. The sterilized test samples exhibited similar delamination compared to non-sterilized samples.

### 3.4. Types of Failure

**Figure 8:**
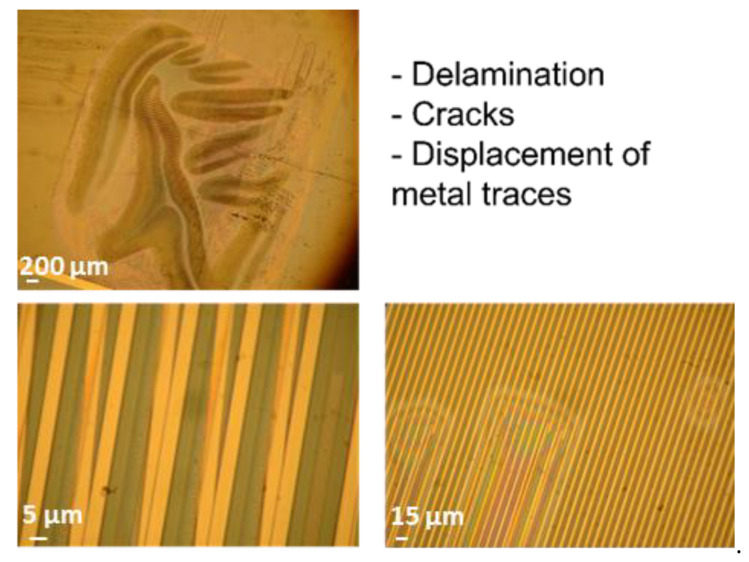
Microscopy images of different failure types of barrel-activated test samples.

**Figure 9:**
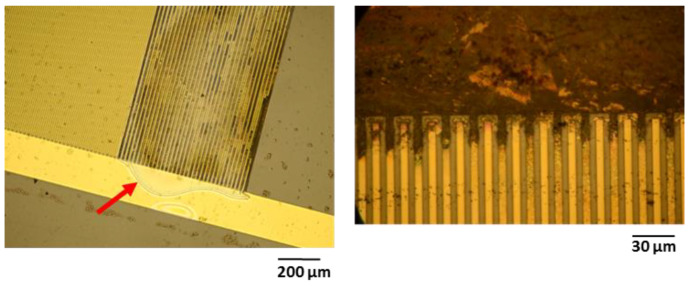
Microscopy images showing the formation of a water film (see red arrow) and the results of the electrochemical reactions of RIE-activated test samples.

The optical investigation of test samples after electrical failure confirmed that barrel-plasma-pretreated samples exhibited a lower adhesion between single layers. For instance, Figure 8 shows that the test samples exhibited large area delamination, displacement of metal traces, and cracks along single metal traces. Such defects can be attributed to low adhesion between the interfaces, which was also demonstrated in this study using cross-cut tests. In contrast to the barrel-plasma-pretreated samples, RIE-pretreated samples did not exhibit such strong delamination between single layers (see Figure 9). These samples did not show any delamination or cracking of the metal feed lines. Here, electrical failure seems to be a result of the PI/metal interface. It is known that the adhesion between PI and Au is not good [22]. Therefore, the formation of wafer films at the PI/metal interface seems to be the origin of the electrical failure of the investigated polymeric encapsulation. As shown in Figure 9, weak adhesion led to the formation of a water film between the large area of Au feed lines and the PI layer (see red arrow). The water film spread out from the feed lines toward the IDE region of the test sample, causing electrochemical reactions and subsequent failure of the device.

## 4. Conclusions and Outlook

In this work, we showed that the adhesion between single layers of neural probes plays a key role in the long-term stability of such devices. We fabricated test samples with different pretreatment methods (barrel plasma and RIE) before spin coating the second encapsulation layer. The stability associated with the different pretreatment methods was investigated using an electrical stress test, mechanical cross-cut tests, and optical microscopy. The electrical stress test (combined with mechanical stressing in the form of ultrasonic treatments) resulted in intact encapsulation behavior of the barrel-pretreated samples for around 1.5 days, whereas RIE-pretreated samples exhibited stable behavior for 24 days. Therefore, RIE-pretreated samples exhibited a 16 times longer intact electrical encapsulation. The cross-cut test showed that the adhesion between single layers of barrel-pretreated samples was lower compared to that in RIE-pretreated samples. Whereas no delamination was observed for RIE pretreated samples, the cross-cut test resulted in total delamination of the top PI foil of barrel-activated samples. Therefore, pretreatment seems to play a key role in the stability of polymeric encapsulations. Optical inspection of the destroyed samples confirmed the differences in mechanical stability. Whereas barrel-plasma-activated samples exhibited delamination of metal traces and the PI layer, RIE activated samples did not show complete delamination of the layers.

Based on the results obtained in this work, it can be concluded that electrical insulation stability is closely related to the adhesion between single layers of the device. An increased surface area may be the result of an RIE pretreatment process and thus result in increased adhesion compared to chemical plasma treatments. However, we demonstrated that RIE pretreatment resulted in a 16 times longer period of stability of the electrical encapsulation compared to chemical-plasma-pretreated samples. Therefore, the adhesion between single layers of a neural probe is crucial for the long-term stability of such devices and needs to be investigated further.

The presented study clearly showed that the adhesion between single thin films plays a key role in the long-term stability of PI-based neural interfaces. The method applied in the present study provides a testing scheme to evaluate the long-term stability in an in vitro environment. Furthermore, the performance of newly developed fabrication schemes can be benchmarked before proceeding to in vivo experiments. In the next step, the long-term stability of adhesion could be further enhanced with the use of adhesion promoters, such as silanes. The functional groups at the PI surface could be used to induce covalent bonding between thin film layers.

## Data Availability

Data are contained within the article.
